# Efficacy of family centered empowerment model of support on care burden in family caregivers of patients with prostate cancer

**DOI:** 10.12669/pjms.41.7.12281

**Published:** 2025-07

**Authors:** Wenhua Jiang, Yuqi Yang, Xiaoping Shi, Wanqing Ni, Xiao Guo, Haiying Cheng

**Affiliations:** 1 Wenhua Jiang, Department of Urology, Jiaxing Second Hospital, Jiaxing, Zhejiang Province 314000, P.R. China; 2 Yuqi Yang, Department of Urology, Jiaxing Second Hospital, Jiaxing, Zhejiang Province 314000, P.R. China; 3 Xiaoping Shi, Department of Urology, Jiaxing Second Hospital, Jiaxing, Zhejiang Province 314000, P.R. China; 4 Wanqing Ni, Department of Urology, Jiaxing Second Hospital, Jiaxing, Zhejiang Province 314000, P.R. China; 5 Xiao Guo, Department of Urology, Jiaxing Second Hospital, Jiaxing, Zhejiang Province 314000, P.R. China; 6 Haiying Cheng, Department of Urology, Jiaxing Second Hospital, Jiaxing, Zhejiang Province 314000, P.R. China

**Keywords:** Caregiving burden, Family caregivers, Family Empowerment Model, Prostate cancer

## Abstract

**Objective:**

Family caregivers of patients with prostate cancer (PCa) are exposed to the increased risk of care burden. This study aimed to determine the effect of the Family Centered Empowerment Model (FCEM) on the care burden of family caregivers of PCa patients.

**Methods:**

This retrospective analysis was conducted at Jiaxing Second Hospital and included data from 120 family caregivers of patients with PCa who were treated from April 2023 to September 2024. The data of 60 family caregivers who received FCEM-based courses were matched in a 1:1 ratio with the data of caregivers who received routine training at the medical center. The two groups were evaluated before and six weeks after intervention using the Caregiver Strain Index (CSI), the Chinese version of the Family Management Scale (FaMM), the Self-Rating Anxiety Scale (SAS) and Self-rating depression scale (SDS), and the World Health Organization Quality of Life Questionnaire (WHOQOL-BREF).

**Results:**

The sample consisted of 59.2% female and 40.8% male caregivers, with an average age of 48.5 years. The pre-intervention CSI, FaMM, SAS, SDS, and WHOQOL-BREF scores were similar in the two groups. After the intervention, both groups reported significantly improved CSI, FaMM, SAS, SDS, and WHOQOL-BREF scores; however, the improvement was considerably higher in the FCEM group of caregivers (P < 0.05).

**Conclusions:**

FCEM program can efficiently reduce stress, anxiety, and depression of family caregivers, which is beneficial for improving their disease management abilities and quality of life (QOL).

## INTRODUCTION

In recent years, the incidence rate of prostate cancer (PCa) has been on the rise, significantly impacting the health and the quality of life (QOL) of patients.^1^,^2^ Currently, the treatment options for PCa include surgery, endocrine therapy, radiotherapy, and chemotherapy.^3^ Since most of the post-treatment care of PCa patients is done by the family members,^4^ lack of accurate and clear understanding of the patient’s illness and nursing-related knowledge often results in stress and affects the physical and mental health of family caregivers, as well as impacts their QOL.^4^-^6^ However, there is currently a lack of training programs to enhance family caregivers’ knowledge of nursing and intervention plans, which can alleviate the care burden.^6^-^8^

The family-centered empowerment model (FCEM) refers to the power granted to patients and their primary caregivers by healthcare professionals to participate in disease management and rehabilitation jointly.^9^,^10^ This model represents a shift from traditional unilateral treatment by medical staff to joint participation by family members, which enhances the caregiving abilities of family members, reduces the care burden, and improves quality of life (QOL).^9^-^10^

FCEM has gradually been applied in clinical interventions for cancer patients. However, due to the small sample size of each study and the lack of relevant evidence-based support, most studies have focused on the experiences of PCa patients or healthcare professionals. This study aimed to explore the effects of FCEM model intervention on the stress, anxiety, depression, management ability, and QOL of family caregivers of PCa patients.

## METHODS

Data from 120 PCa patient family caregivers collected between April 2023 to September 2024 were retrospectively analyzed. The sample size for this study was calculated based on the expected effect size of similar interventions. A power analysis was performed using G^*^Power software, with a significance level of 0.05 and a power of 0.80. The analysis indicated that 120 participants were sufficient to detect meaningful differences in caregiver burden outcomes, assuming a medium effect size (Cohen’s d = 0.5). The FCEM group consisted of 60 family caregivers who received FCEM-based courses, while the control group consisted of 60 caregivers who received routine training at the medical center.

### Ethical approval:

This study was conducted with the approval of the Medical Ethics Committee of Jiaxing Second Hospital (Approval number: 2023-ZFYJ-016-01; dated: March 20, 2023).

### Inclusion criteria:

- Qualification for nursing PCa patients (For example, as a family member primarily responsible for providing daily care to patients, providing at least eight hours of daily care and living with patients).
- Age range: 18-60 years old.
- Communication devices such as mobile phones, personal computers, and tablets equipped with chat software (such as WeChat, QQ, etc.) are available.
- Reading and writing skills, literacy ability.
- Not participated in any courses similar to family training (research projects).
- No experience working as a member of a medical team.

### Exclusion criteria:

- Absent from more than one training course.
- Family caregivers with medical issues, including physical, mental, and psychological illnesses.
- Caregivers who do not cooperate in completing all questionnaire assessments.

The routine training content in medical centers included explaining the basic knowledge of PCa, treatment measures, daily precautions related to the condition, nursing measures, and prevention of complications. Close communication with the patient’s family caregivers was maintained through social media. The medical center team promptly assessed patients’ progress and treatment status, imparted relevant care skills, and patiently answered questions from family caregivers.

The FCEM-based course was divided into four steps: self-efficacy, problem-solving, enhancing self-esteem, and summative assessment. The course was divided into four training sessions. Initially, the educational content of online courses was developed through checklists tailored to the needs of research institutions. Then, FCEM was presented as an online group discussion, divided into 10 groups of six people each. The content was presented in four sessions, each lasting 40-60 minutes, through social media. The implementation steps are summarized in Table-I.

**Table-I:** FCEM based intervention methods.

Topics	Content of sessions	Objectives of sessions	Presentation method
First Course (Perceived Threat)	Raise awareness of the importance of nursing staff and families in controlling PCa and caring for patients Be familiar with the importance and sensitivity of PCa.	Introducing the importance and status of nursing staff in providing health care for PCa patients. 2. Teaching the importance of knowledge and skills related to PCa and sensitivity to PCa prognosis and treatment.	1. Lectures, Q&A sessions, group discussions, and sharing of nursing staff experiences. 2. Presentation (PowerPoint PPT). 3. Education manual or video.
Second Course (Self Efficacy)	1. Understanding drug therapy and its role in preventing recurrence, be familiar with drug therapy, complications, and nursing. 2. Familiarizing oneself with stress coping strategies, effective communication with patients, problem-solving methods, time management, and the ability to cope with the role of nursing caregiver	1. Improving knowledge and skills in drug therapy. 2. Improving the preparation for nursing PCa patients. 3. Improving communication skills with PCa patients and adapting to patient care. 4. Improving the ability to reduce stress and nursing burden.	1. Lectures, Q&A sessions, group discussions, and sharing of nursing staff experiences. 2. Education manuals or videos. 3. Online consultation and telephone follow-up.
Third Course ( Self Esteem)	Building confidence in how to care for and handle disease symptoms, referral conditions, and procedures in the event of an outbreak.	1. Improving trust in improving patient care readiness. 2. The confidence and control of nursing caregiver in patient care.	1. Lectures, Q&A sessions, group discussions, and sharing of nursing staff experiences. 2. Education manuals or videos. 3. Online consultation and telephone follow-up.
Fourth Course (Evaluation)	Summarizing the content of the previous classes and completing the questionnaire on stress, disease management, anxiety and depression, and QOL.		Lectures, Q&A sessions

***Note:*** FCEM, Family Empowerment Model.

The intervention consisted of four modules, each designed to enhance caregivers’ abilities and emotional support in different aspects. First, the Self-Efficacy Module aimed to increase caregivers’ confidence in managing patient care and handling caregiving stress, with training on stress management techniques, time management, and problem-solving skills. Next, the Psychological Health Module focused on caregivers’ emotional well-being, offering strategies for managing anxiety and depression, such as mindfulness practices and emotional support techniques. The Self-Esteem Module aimed to boost caregivers’ self-esteem by helping them recognize their strengths and the value of their role in caregiving. Finally, the Evaluation Module included a review of key concepts and an assessment of caregivers’ skills, reinforcing the learned material and evaluating the effectiveness of the training.

The online component of the intervention was delivered via WeChat, a widely used social media platform in China. Caregivers participated in weekly group discussions, where they could share experiences, ask questions, and receive feedback from facilitators. Additionally, caregivers were encouraged to communicate with each other between sessions through a dedicated WeChat group, allowing for continuous peer support. Follow-up consultations via WeChat were provided to address individual concerns and ensure caregivers felt supported throughout the intervention.

### Data collection:

Data collection tools, including demographic information questionnaires, The Caregiver Strain Index (CSI), Family Management Measure (FaMM), Self-Rating Anxiety Scale (SAS), Self-Rating Depression Scale (SDS), and World Health Organization QOL Questionnaire (WHOQOL-BREF), were completed before and six weeks after the intervention. Demographic information included age, gender, marital status, education level, patient relationship, and place of residence. CSI consisted of 13 items, scoring One for Yes and 0 for No.` A higher score indicated more significant caregiver pressure. The FaMM scale was divided into the Family Difficulty Subscale (14 items in total) and the Family Management Ability Subscale (12 items in total), both of which were evaluated using a 5-point scoring method.

The Caregiver Strain Index (CSI) measures various aspects of caregiving burden, including emotional, physical, and social strain. The emotional strain subscale assesses the caregiver’s feelings of anxiety and frustration, while the physical strain subscale evaluates the caregiver’s physical exhaustion and the burden of daily caregiving tasks. The social strain subscale measures the impact of caregiving on the caregiver’s social relationships and activities. The CSI results were correlated with the Self-Rating Anxiety Scale (SAS), Self-Rating Depression Scale (SDS), and the WHOQOL-BREF, which assess psychological health and quality of life.

The Self-Rating Anxiety Scale (SAS), Self-Rating Depression Scale (SDS), and World Health Organization Quality of Life Questionnaire (WHOQOL-BREF) have been widely validated and shown to be reliable instruments for assessing caregiver stress and well-being. Previous studies have demonstrated their internal consistency and construct validity in different populations.^11^ These scales have been validated in Chinese populations, with Cronbach’s alpha values greater than 0.70 for each instrument.

A higher score on the family difficulty subscale indicated greater difficulty in managing the disease. A higher score of the subscale for family management ability indicated stronger family management ability. The Self-Rating Anxiety and Depression Scale (SAS, SDS) included 20 items reflecting subjective feelings in the past week, with each item rated on a scale of 1-4. The total scale score was the sum of the scores of each item. The standard was divided into coarse fractions and then multiplied by the integer part of 1.25. According to the Chinese norm, scores greater than 50 indicate the presence of anxiety, with higher scores indicating more severe anxiety. The SDS contained 20 items that reflected subjective feelings, physical symptoms, psychomotor disorders, and psychological disorders experienced in the past week. Each item was scored on a scale of 1-4, and the total score was the sum of the scores for each item. The standard was divided into coarse fractions and multiplied by the integer part 1.25. According to the Chinese norm, scores greater than 53 indicate the presence of depression, with higher scores indicating more severe depression. The WHOQOL-BREF included social functioning, emotional state, and physical functioning, scoring 100 points for each dimension. The higher the score, the better the QOL.

### Statistical analysis:

SPSS 25.0 software was used for the statistical analysis. According to the Shapiro-Wilk test for assessing distribution normality, continuous variables were reported as mean and standard deviation (SD) or median and interquartile range (IQR). Normal distribution data were represented by mean ± SD. An independent sample t-test was used to compare two groups, and a paired t-test was used for comparison within the same group. Non-normally distributed data are represented by median and interquartile range. Mann-Whitney U test was used to compare two groups, and Wilcoxon signed-rank test was used for comparison within the same group. The chi-square test represented count data by frequency and composition ratio (%). The statistical analysis results were defined as P < 0.05, indicating a statistically significant difference.

## RESULTS

A total of 120 family caregivers participated in this study, comprising 53 males and 67 females. The age range was 27-59 years, with a median age of 49. The FCEM group (n = 60) was matched with a control group in a 1:1 ratio. There was no statistically significant difference in basic characteristics such as age, gender, patient relationship, marital status, education level, and place of residence between the two groups (P>0.05), Table-II.

**Table-II:** Demographic variables of PCa patients’ family caregivers.

Variables		FCEM group (n=60)	Control group (n=60)	Z/χ^2^	P
Age (year), M(P25/P75)		48 (43-53.5)	51.5 (45-56)	-1.157	0.247
Sex, n (%)	Male	25 (41.7)	28(46.7)	0.304	0581
	Female	35 (58.3)	32 (53.3)		
Relationship with patients, n (%)	Spouse	28 (46.7)	37 (61.7)	3.138	0.208
	Children	21 (35.0)	17 (28.3)		
	Other	11 (18.3)	6 (10.0)		
Marital status,n (%)	Married	46 (76.7)	53 (88.3)	2.828	0.093
	Unmarried	14 (23.3)	7 (11.7)		
Educational level, n (%)	Primary school	16 (26.7)	17 (28.3)	2.051	0.359
	Middle school	30 (50.0)	35 (58.3)		
	High school and above	14 (23.3)	8 (13.3)		
Residence, n (%)	Rural area	20 (33.3)	16 (26.7)	0.635	0.426
	Town	40 (66.7)	44 (73.3)		

***Note:*** FCEM, Family Empowerment Model; PCa, prostate cancer.

**Fig.1:**
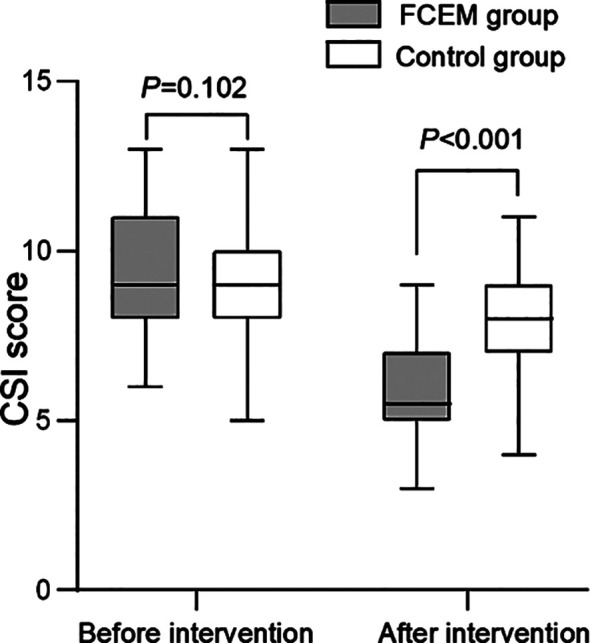
Comparison of CSI scores between two groups before and after intervention. FCEM, Family Empowerment Model; CSI, Caregiver Strain Index.

There was no statistically significant difference in CSI scores between the FCEM and the control groups before intervention (P=0.102). After the intervention, the CSI score of both groups significantly decreased and was considerably lower in the FCEM group than the control group (P<0.001) (Fig.1).

Before intervention, there was no significant intergroup difference FaMM scores. Table-III After intervention, both groups demonstrated substantial differences in scores compared to before intervention (P < 0.001); the FCEM was associated with a significantly lower family difficulty subscale score (34.3 ± 5.0) compared to the control group (37.8 ± 6.0) (P = 0.001). In contrast, the family management ability subscale score (48.0 ± 5.6) of the FCEM group was significantly higher than that of the control group (44.2 ± 6.3) (P = 0.001).

**Table-III:** Comparison of the FaMM scores between two groups (Mean ± SD, scores).

Variables		FCEM group (n=60)	Control group (n=60)	t	P
Family difficulty	Before intervention	43.4±6.0	42.7±6.2	0.641	0.523
	After intervention	34.3±5.0*	37.8±6.0*	-3.545	0.001
Family management ability	Before intervention	38.3±4.3	37.0±5.2	1.556	0.122
	After intervention	48.0±5.6*	44.2±6.3*	3.421	0.001

***Note:*** FCEM, Family Empowerment Model; FaMM, Family Management Measure. Compared with before treatment in the same group,

* P<0.05.

The pre-intervention SAS (53.0 ± 6.3) and SDS scores (56.7 ± 4.8) were comparable in the FCEM and control groups (51.8 ± 5.2 and 55.7 ± 5.4, respectively) (P = 0.244 and P = 0.284, respectively). After the intervention, both scores were significantly reduced in the two groups and were substantially lower in the FCEM group than those in the control group (P<0.001) (Table-IV).

**Table-IV:** Comparison of SAS and SDS scores between two groups (Mean ± SD, scores).

Variables		FCEM group (n=60)	Control group (n=60)	t	P
SAS	Before intervention	53.0±6.3	51.8±5.2	1.17	0.244
	After intervention	41.9±4.8*	46.8±5.3*	-5.329	<0.001
SDS	Before intervention	56.7±4.8	55.7±5.4	1.075	0.284
	After intervention	41.5±4.5*	48.3±4.1*	-8.638	<0.001

***Note:*** FCEM, Family Empowerment Model; Compared with before treatment in the same group,

* P<0.05.

Similarly, no significant intergroup difference in the scores of social relationships, psychological, and physiological domains was reported before the intervention (P>0.05). After the intervention, the scores of the above variables increased significantly in both groups and were considerably (P<0.001) higher in the FCEM group [79 (77-84), 82.2 ± 5.4, 82 (76-84)] compared to the control group [74 (72-77), 78.5 ± 6.7, 75.5 (73-80)] (Table-V).

**Table-V:** Comparison of WHOQOL-BREF scores between two groups (Mean ± SD, scores).

Variables		FCEM group (n=60)	Control group (n=60)	Z/t	P
Social relationships	Before intervention	64.5 (61-68)	65 (62-69)	-1.248	0.212
	After intervention	79 (77-84)*	74 (72-77)*	-5.127	<0.001
Psychological health	Before intervention	62.3±4.8	60.8±6.0	1.494	0.138
	After intervention	82.2±5.4*	78.5±6.7*	3.31	0.001
Physical health	Before intervention	72 (68-77.5)	70 (65-78.5)	-1.243	0.214
	After intervention	82 (76-84)*	75.5 (73-80)*	-3.636	<0.001

***Note:*** FCEM, Family Empowerment Model; WHOQOL-BREF, World Health Organization QOL Questionnaire; Compared with before treatment in the same group,

* P<0.05.

## DISCUSSION

This study aimed to investigate the effects of FCEM on stress, disease management, anxiety and depression, and QOL of family caregivers of PCa patients. The results showed that compared with routine training in medical centers, the ECEM-based intervention program was more beneficial in reducing care pressure, improving disease management ability, alleviating negative emotions, and improving the QOL of the caregivers. Several factors may explain the stronger effects of the FCEM intervention. The model emphasizes active participation, personalized education, and emotional support, which may lead to greater caregiver engagement and skill acquisition. Unlike routine training that focuses mainly on disease knowledge, FCEM sessions involve interactive elements like group discussions, scenario-based learning, and peer feedback. These components may enhance caregivers’ sense of control, increase their self-efficacy, and help them better manage stress and psychological burden. Furthermore, the inclusion of continuous online support may have reinforced behavior change and emotional resilience.

The results of this study indicate that interventions based on FCEM significantly reduce the nursing burden on family caregivers of PCa patients. This is consistent with other research findings. Previous research has shown that the FCEM program has a substantial impact on the nursing burden and self-efficacy of caregivers for hemodialysis patients.^12^ Due to the feasibility, simplicity, and practicality of the intervention, the FCEM-based intervention plan reduces the stress, anxiety, and depression of the nursing staff.10 However, according to the study by Mousaei et al., FCEM did not show significant effects in reducing the burden of COVID-19 care.^13^ This may be due to differences in sample size, varying time points, or participant background heterogeneity.

Empowerment interventions emphasize the dominant role of family caregivers, providing them with more power and resources to address various issues in the caregiving process.^13^,^14^ Together with previous reports, this study further proves that providing relevant disease knowledge training improves the ability of caregivers to understand the development and nursing needs of PCa patients,^13^-^15^ allowing them to be more adept in the care process. This, in turn, reduces anxiety and stress caused by a lack of understanding of the condition.^16^

Many family caregivers may have concerns about the impact of caring for PCa patients on their daily lives.^17^ While most family caregivers possess a certain amount of essential knowledge about the disease, they often struggle to apply this knowledge in practical management.18 Therefore, providing disease management skills for family caregivers is crucial.^18^,^19^ The results of this study indicate that FCEM intervention offers unique advantages in enhancing caregivers’ disease management capabilities. In addition to teaching caregivers how to perform basic nursing operations, FCEM-based interventions provide more systematic and comprehensive disease management training, including assisting patients with medication administration and conducting simple rehabilitation therapies.^20^ More importantly, FCEM-based interventions are instrumental in cultivating caregivers’ disease management thinking. Caregivers can learn to develop personalized management plans tailored to the patient’s specific situation, promptly identify changes in the patient’s condition, and take corresponding measures.20,21 In contrast, routine care lacks such deep and personalized interventions, which limits caregivers’ ability to improve their disease management skills.

This study found that FCEM intervention has a more positive effect on alleviating caregivers’ anxiety and depression. The FCEM plan developed in this study is continuous. In addition to face-to-face education, online nursing groups were established on WeChat social media, where family caregivers could consult and share positive information when encountering problems, which is crucial for reducing anxiety and depression among family caregivers. Moreover, home caregivers often feel anxiety because they lack professional nursing knowledge.^20^-^22^ Since caring for PCa patients takes up most of the caregiver’s leisure and social activity time, negative emotions such as anxiety and depression increase, thereby reducing the caregiver’s QOL. The results of this study indicate that the intervention based on FCEM has significant implications for improving the QOL of caregivers. These results are consistent with previous research. Higher caregiver self-efficacy is associated with a lower risk of adverse outcomes.^23^ The second course of FCEM-based intervention in this study focused on improving caregivers’ self-efficacy and health literacy, thus reducing the burden of nursing and negative emotions.

The results of this study demonstrate the positive role of FCEM-based intervention for family caregivers of PCa patients. This intervention model provides an effective nursing approach for improving family caregivers and helps to build a more harmonious and healthy family care environment.

### Strengths of the study:

This study has several strengths. It is among the first to evaluate the impact of a structured FCEM intervention on caregivers of prostate cancer patients in a Chinese context. The use of multiple validated psychological and quality-of-life measures adds robustness to the findings. Moreover, the blended approach combining face-to-face and online components increases the intervention’s feasibility.

### Limitations:

Firstly, it was a small-sample, single-center study. Although a matched cohort was used, the retrospective design and small sample size limit the strength of conclusions about the effectiveness of FCEM in reducing caregiver burden. Caregivers also vary in their learning abilities and in their capacity to apply educational content to patient care. Additionally, potential bias may exist in assessing caregivers’ psychological health status. The follow-up occurred only six weeks after the intervention, which may not adequately capture the long-term effects of FCEM. The single-center design of the study further limits the generalizability of the findings. Implementing FCEM in different healthcare systems may face challenges such as differences in healthcare infrastructure, caregiver literacy levels, and cultural perceptions of caregiving. For instance, in rural areas, limited access to online platforms and lower levels of formal education among caregivers could affect the effectiveness of the intervention. Future studies should consider expanding the sample size, including hospitals from diverse regions and healthcare levels, and exploring how FCEM can be adapted to various settings and populations to improve the generalizability and practical value of the findings.

## CONCLUSION

The results of this study indicate that FCEM can significantly reduce the burden on family caregivers of patients with PCa, alleviate negative emotions, and improve disease management ability and quality of life (QOL). FCEM seems to be able to help caregivers improve the quality of home healthcare, as it is efficient, affordable, and safe. However, further research is necessary to explore the mechanisms by which FCEM affects depression, anxiety, and stress among family caregivers.

### Authors’ contributions:

**WJ:** Literature search, study design and manuscript writing. manuscript revision and validation and is responsible for the integrity of the study **YY, XS, WN, XG and HC:** Data collection, data analysis and interpretation. Critical Review. All authors have read and approved the final manuscript.
